# Residual Structure of *Streptococcus mutans* Biofilm following Complete Disinfection Favors Secondary Bacterial Adhesion and Biofilm Re-Development

**DOI:** 10.1371/journal.pone.0116647

**Published:** 2015-01-30

**Authors:** Tatsuya Ohsumi, Shoji Takenaka, Rika Wakamatsu, Yuuki Sakaue, Naoki Narisawa, Hidenobu Senpuku, Hayato Ohshima, Yutaka Terao, Takashi Okiji

**Affiliations:** 1 Division of Cariology, Operative Dentistry and Endodontics, Department of Oral Health Science, Niigata University Graduate School of Medical and Dental Sciences, Niigata, Japan; 2 Department of Food Bioscience and Biotechnology, College of Bioresource Sciences, Nihon University, Kanagawa, Japan; 3 Department of Bacteriology I, National Institute of Infectious Diseases, Tokyo, Japan; 4 Division of Anatomy and Cell Biology of the Hard Tissue, Department of Tissue Regeneration and Reconstruction, Niigata University Graduate School of Medical and Dental Sciences, Niigata, Japan; 5 Division of Microbiology and Infectious Diseases, Department of Oral Health Science, Niigata University Graduate School of Medical and Dental Sciences, Niigata, Japan; Virginia Commonwealth University, UNITED STATES

## Abstract

Chemical disinfection of oral biofilms often leaves biofilm structures intact. This study aimed to examine whether the residual structure promotes secondary bacterial adhesion. *Streptococcus mutans* biofilms generated on resin-composite disks in a rotating disc reactor were disinfected completely with 70% isopropyl alcohol, and were again cultured in the same reactor after resupplying with the same bacterial solution. Specimens were subjected to fluorescence confocal laser scanning microscopy, viable cell counts and PCR-Invader assay in order to observe and quantify secondarily adhered cells. Fluorescence microscopic analysis, particularly after longitudinal cryosectioning, demonstrated stratified patterns of viable cells on the disinfected biofilm structure. Viable cell counts of test specimens were significantly higher than those of controls, and increased according to the amount of residual structure and culture period. Linear regression analysis exhibited a high correlation between viable and total cell counts. It was concluded that disinfected biofilm structures favored secondary bacterial adhesion.

## Introduction

The control and removal of dental biofilms are primary aims in the prevention of dental caries and periodontal disease. Mechanical removal of biofilm using a toothbrush and dental floss is recommended as a major part of oral hygiene care. A wide range of antimicrobial agents, such as stannous fluoride, sodium fluoride, triclosan, chlorhexidine digluconate, quaternary ammonium compounds, surfactants, enzymes and metal ions, have been formulated into oral care products in order to enhance the effects of mechanical plaque control [1–5]. Systemic or topical application of antimicrobials is also used as an alternative or adjunctive method, mainly for reasons of limited access [6–8]. It has been demonstrated that chemical control using antimicrobial compounds provides some antimicrobial benefit and improves clinical parameters, including plaque index and gingival inflammation [4, 6, 8]. In addition, attempts have been made to incorporate antimicrobial compounds into dental materials, such as acrylic resins, in order to control surface biofilm formation [9, 10].

Previous studies have focused mainly on how microorganisms could be rapidly killed using high concentrations of antimicrobials or by new antimicrobial compounds [11]. However, there are some concerns that bacteria may continue to develop resistance to currently available antimicrobial agents [11]. Moreover, recent investigations have reported several limitations to chemical disinfections for oral biofilms [12–17]. Some reports have demonstrated that antimicrobial compounds do not work as intended [12, 16, 17]. This phenomenon can be explained as follows: (1) reduced penetration of the agent due to degradation and/or modification by the biofilm matrix [18–23]; (2) alteration of metabolic activity as a stress response [19, 24]; and (3) existence of tolerant or dormant cells [25, 26]. Another limitation is that a single use of chemical compounds without mechanical removal may leave intact biofilm structures, even after the eradication of microorganisms [14, 16, 17, 27]. In line with this notion is the finding that treatment of *Porphyromonas gingivalis* biofilms with chlorhexidine gluconate (CHX) for 5 min does not degrade their external structure, or reduce the volumes of protein and carbohydrate constituents [28]. As the remaining biofilm matrix contains carbohydrates, proteins, polysaccharide, lipids and nucleic acid, dead bacteria and biofilm components could work as antigens and induce host inflammatory reactions [29–31]. The residual structure is a source of calculus formation [32, 33], and may serve as an ideal substrate to promote new microbial adhesion and biofilm re-formation. In the present study, we tested whether the residual structure of disinfected biofilms promotes bacterial secondary adhesion and re-development using an *in vitro* oral biofilm model.

## Materials and Methods

### Preparation of residual biofilm structure

*Streptococcus mutans* ATCC 25175 (serotype c), which was isolated from carious dentin, was purchased from American Type Culture Collection, and was grown in brain heart infusion broth (BHI; Difco Laboratories, Detroit, MI) at 37°C under anaerobic conditions. Starter culture was transferred into 10 ml of fresh BHI and grown for 4 h at 37°C under aerobic conditions. Optical density at 600 nm (OD_600_) of all bacterial suspensions was adjusted to 0.05 prior to inoculation.

A resin composite material (Premise Flowable, Kerr, Orange, CA) was used as an adhering site to prepare residual biofilm structure. Standardized disks, 6 mm in diameter and 1.5 mm in thickness, were prepared and polished with 4000 grit waterproof silicon carbide paper, and subjected to ethylene oxide gas sterilization for 4 h. Specimens were coated with 10% sterile saliva for 2 h at room temperature. A sterile saliva solution was prepared as described previously [34]. Unstimulated saliva was obtained from one healthy person (one of the authors). Saliva samples were diluted (1:10) with sterile Ringer solution containing 0.05% cysteine (Sigma Aldrich, St Louis, MO). Diluted solution was then centrifuged for 10 min and the supernatant was filter sterilized.

*S. mutans* biofilms were developed on the disks using a rotating disk biofilm reactor (RDR; Biosurface Technologies Corp., Bozeman, MT). This system has been described in detail previously [35]. The disk was initially incubated for 90 min at 37°C in BHI broth containing a standard cell suspension with stirring at 75 rpm to achieve initial adhesion [36]. Following the adhesion phase, the stir disk was gently rinsed in 100 ml of phosphate buffer (5.0 g l^−1^ NaCl and 2.5 g l^−1^ Na_2_HPO_4_; pH 7.4), and was aseptically transferred to a sterile reactor vessel filled with 300 ml of 1:10 strength BHI broth containing 0.05% sucrose. The biofilm was allowed to develop for 24 or 72 h with stirring at 50 rpm under continuous-flow aerobic conditions at a rate of 4.6 ml min^−1^ during incubation at 37°C. After the fixed incubation period, the rotating wheel was aseptically removed, and specimens were washed three times with phosphate buffer, followed by immersion in 70% isopropyl alcohol for 120 min for complete disinfection of microorganisms in the biofilm. Complete absence of viable bacteria after isopropyl alcohol treatment was confirmed by the culture method.

### Secondary bacterial adhesion and biofilm re-development

Disinfected biofilm structures on resin composites were washed three times with phosphate buffer, and were then returned to a new sterile reactor vessel filled with 300 ml of the bacterial solution in the logarithmic phase (OD_600_ = 0.05). The 1/10 strength BHI broth containing 0.05% sucrose was pumped into the reactor and the microorganisms were allowed to develop for 15 min or 4 h. The optical density in the vessel was maintained up to 4 h by adjusting the flow rate. Disks without structures and disinfected biofilm alone served as controls. Finally, the rotating disk was aseptically removed and immersed in sterile phosphate buffer in order to remove non-adherent cells, and specimens were gently removed for further investigation.

### Fluorescent staining and confocal laser scanning microscopic observation

Following the secondary streptococcal adhesion phase, specimens were stained with calcein-AM (Molecular Probes/Invitrogen, Eugene, OR) for live cells and rhodamine-B (Wako Pure Chemical Industries Ltd., Osaka, Japan) for the whole structure. Calcein-AM diffuses passively into the cytoplasm, where it is converted into green-fluorescent calcein via native esterases. Calcein fluorescence is retained in live cells until plasma membrane permeability is compromised [14, 16, 17]. Rhodamine-B is a counterstaining dye, revealing the extent of biomass, independent of its activity [37]. Biofilm specimens were stained with 10 μg ml^−1^ calcein-AM in phosphate buffer for 2 h at 37°C, followed by staining with 5 μg ml^−1^ rhodamine-B for 5 min. A biofilm structure without the secondary adhesion phase served as a control.

Biofilms were imaged on a confocal laser scanning microscope (CLSM; FV-300, Olympus, Tokyo, Japan) using Ar 488-nm and He-Ne 543-nm lasers. Filters were set to 510–530 nm for the detection of calcein-AM and above 610 nm for rhodamine-B. A ×60 water-immersion objective lens was used for direct observation. Stacks of fluorescent images were collected in the z-dimension, and three-dimensional reconstruction was carried out using Imaris software (Bitplane AG, Zurich, Switzerland).

### Cryoembedding, cryosectioning and quantitative analysis

Fluorescent stained biofilm samples were embedded with Tissue-Tek O.C.T. compound (Sakura Finetek, Tokyo, Japan), as described previously [37]. The composite was placed on dry ice, and the medium was allowed to freeze. Biofilm was removed from the composite, and then the biofilm with the embedded side down was placed on dry ice. The embedding medium was then used to cover the exposed biofilm bottom surface. The embedded sample was sectioned into 8-μm cross sections in a cryostat (CM 3050 S; Leica, Nussloch, Germany). Sections at ten-section intervals were selected for quantitative analysis. The number of sections analyzed was twenty per embedded sample. Analysis was performed with a total of six composites, i.e., one hundred and twenty sections were analyzed.

In order to quantify the ratio of attached viable bacterial cells to a disinfected structure, the total number of green and red pixels of the samples after the secondary adhesion phase for 4 h was enumerated using MetaMorph software (Molecular Devices, Sunnyvale, CA). All image analyses were performed by a single trained investigator.

### Viable and total cell counts

Viable cell counts and PCR-Invader assay were performed in order to quantify viable and total bacteria of samples prepared under various conditions. Samples were washed three times with phosphate buffered saline and placed in 3 ml of phosphate buffered saline. Biofilms were detached by shaking vigorously for 3 min, ultrasonicated for 5 min, and shaken again for 3 min. Bacterial suspensions were homogenized and then divided into equal volumes for viable and total cell count analysis.

For viable counts, suspensions were serially diluted, plated on BHI agar, and incubated anaerobically for 48 h at 37°C.

Quantitative analysis of the total bacterial count was performed using the modified Invader PLUS assay (BML, Inc., Tokyo, Japan) [38]. The Invader PLUS assay is a sensitive, rapid method for the detection and quantification of nucleic acid, showing significant correlations with real-time polymerase chain reaction, with a detection limit of 3.7 log copies [39]. A pair of primers was designed based on a region of the 16S rRNA gene [40]. Bacterial DNA was extracted using Pure LC (Roche, Tokyo, Japan) and MagNA Pure LC Total Nucleic Acid Isolation Kit (Roche). Template DNA (3 ml) was added to 12 ml of reaction mixture containing 20 mM primers, 2.5 mM dNTP, 2.5 U AmpliTaq gold, 3.5 mM primary probe, 0.35 mM Invader oligo, and Invader core reagent kit, which consisted of FRET mix and enzyme/MgCl_2_ solution (F-primer, 5′-GGATTCGCTAGTAATCG-3′; R-primer, 5′-TACCTTGTTACGACTT-3′; Tb-Primary probe, 5′-CGCGCCGAGGCCGGGAACGTATTCACC-3′; Tb-Invader oligo, 5′-TGACGGGCGGTGTGTACAAGGCA-3′). Reaction mixtures were preheated at 95°C for 20 min, and then two-step PCR was carried out for 35 cycles (95°C for 1 s, 63°C for 1 min) using the ABI PRISM 7900 sequence detection system (Applied Biosystems, Foster City, CA). Fluorescence values for FAM (carboxyfluorescein) (wavelength/bandwidth: excitation, 485/20 nm; emission, 530/25 nm) were measured at the end of the incubation/extension step at 63°C for each cycle. Each assay was run in triplicate and mean values from six independent samples were determined.

### Statistical analysis

Statistical analyses were performed using SPSS 11.0 (IBM, Armonk, NY). Where applicable, data are presented as means ± standard deviation (SD). Correlations between secondary biofilm and residual biofilm were evaluated by least-squares curve-fitting analysis. Significance of viable and total cell counts was determined by ANOVA, together with Tukey’s test for the 15-min secondary adhesion phase, and by Student *t*-test at 4 h.

## Results

### CLSM observation and analysis

**Figure 1:**
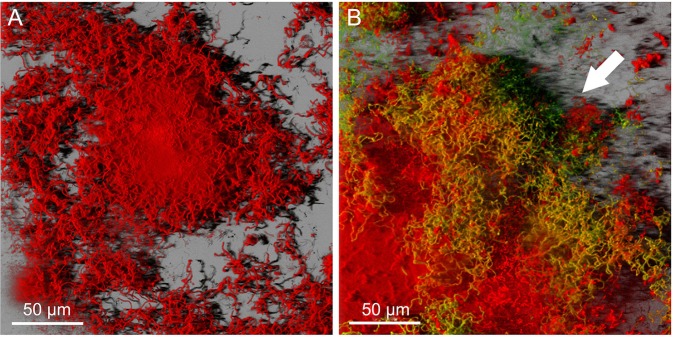
Three-dimensional reconstructed images of *S. mutans* biofilm stained with calcein-AM and rhodamine-B. Three-dimensional reconstructed images of disinfected 72-h structure (A: control) and 4-h secondary biofilm on 72-h structure (B). Fresh planktonic *S. mutans* cells flowed into the completely disinfected 72-h biofilm structure for 4 h. Viable bacteria were stained green by calcein fluorescence (white arrow), and appeared to get caught in upstream edges of disinfected biofilm clusters.

The maximum thickness of residual biofilm structure in this study was approximately 30 μm for 24 h and 80 μm for 72 h. A three-dimensional reconstructed image of the 72-h structure following isopropyl alcohol treatment is shown in Fig. 1A. The image shows that the structure is stained completely red, indicating that isopropyl alcohol treatment resulted in complete absence of viable bacteria.

*S. mutans*, which were pumped into the reactor for 15 min or 4 h in the secondary adhesion phase, adhered to the residual structure and smooth surface on a resin composite under continuous flow conditions. It was observed that the microorganisms tended to adhere to the structured surface. Three-dimensional reconstructed images revealed that viable bacteria appeared to get caught in upstream edges of the disinfected biofilm clusters (Fig. 1B).

**Figure 2:**
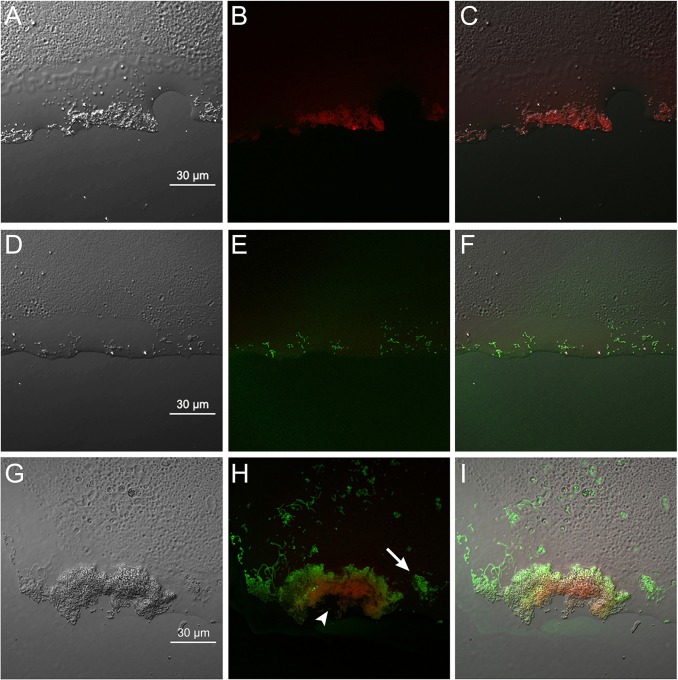
Cryosectioned images of *S. mutans* biofilm stained with calcein-AM and rhodamine-B. Cryosectioned images of disinfected 72-h structure (A to C); 4-h secondary biofilm on a disk without a structure (D to F); and 4-h secondary biofilm on a 72-h structure (G to I). Biofilm was stained with calcein-AM and rhodamine-B (B, E, H). Calcein-AM (green images) indicates that the *S. mutans* cells retained membrane integrity, i.e., live bacterial cells. Rhodamine-B (as red images) is a counterstaining dye, revealing the extent of the biomass, independent of its activity. (A, D, G) Transmission images. (B, E, H) Fluorescent images. (C, F, I) Composite images. In each image, the resin composite interface of the biofilm is on the bottom and the upper layer interface is on the top. Secondary adherent *S. mutans* cells (green images in H and I) have been deposited on the disinfected biofilm structure (red images in H and I; arrowhead) to a greater degree than on the surface without the structure (arrow). Scale bar = 30 μm.

Cryosectioned images (Fig. 2) provided higher resolution of the deeper layers. Disinfected 72-h structure showed no calcein-AM-positive cells (Fig. 2B and 2C), indicating that no microorganisms in the biofilm were viable. The amount of the secondary adherent *S. mutans* cells (as green images) on the disk without a structure (Fig. 2E and 2F) was less than that on the 72-h structure (Fig. 2H and 2I). Viable cells on the disinfected biofilm structure showed a stratified pattern (Fig. 2H and 2I). The percentage (calcein-AM/rhodamine-B) of attached viable bacterial cells was 20.1 ± 14.4% after 4 h of secondary biofilm formation on 72-h disinfected structure, 0% for the disinfected 72-h structure alone and 100% for the control (the disk without a structure).

### Viable and total cell counts

**Figure 3:**
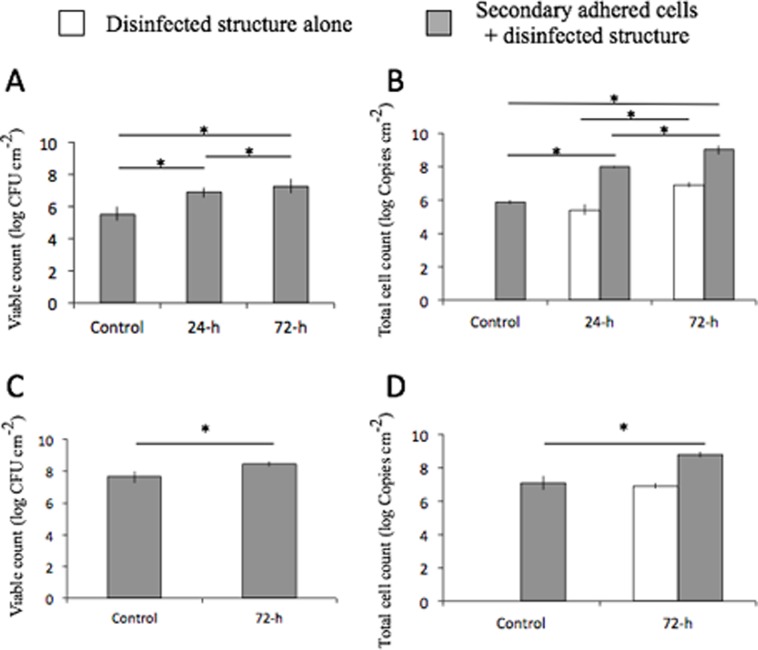
Viable and total cell counts after 15 min and 4 h of secondary adhesion phase on control and disinfected biofilm structures. Viable (A) and total cell counts (B) after secondary adhesion phase for 15 min and viable (C) and total cell counts (D) for 4 h on control (no biofilm structure) and disinfected biofilm structures (24-h and 72-h). Data are presented as means ± SD (n = 6). Secondary bacterial adhesion significantly increased in the presence of a residual structure in comparison with the control group (ANOVA with Tukey’s test, *p<0.05).

Viable and total cell counts after 15 min of secondary adhesion phase on control (no biofilm structure) and disinfected structures (24-h and 72-h) are shown in Fig. 3A and B. Rank order of counts was as follows: 72-h group > 24-h group > control. There were significant differences among the three groups (p<0.05). The disinfected structure increased with time (Fig. 3B, open bar), allowing to develop bacterial secondary adhesion. Fig. 3C and D show viable and total cell counts after 4 h of secondary adhesion phase on control and disinfected structures (72-h). Both counts in the 72-h group were significantly higher than those in controls (p<0.05).

**Figure 4:**
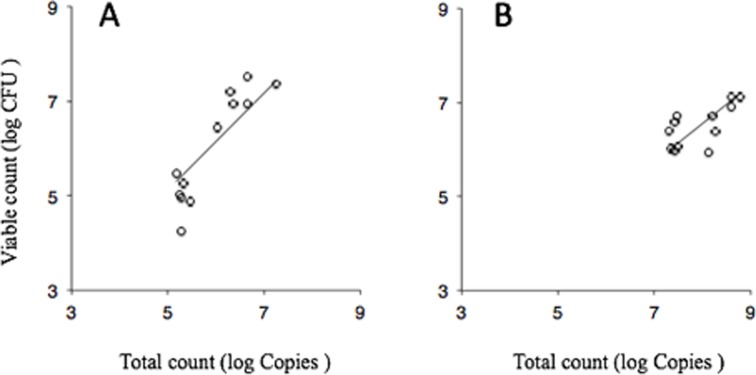
Least-squares curve-fitting analysis between total counts and viable counts. Correlation between PCR-Invader-derived total counts and viable counts after flow for 15 min or 4 h on control (A; no biofilm structure) and disinfected biofilm structures (B). Solid line is derived from a least-squares curve-fitting analysis. Data for controls in Fig. 3 are plotted in Fig. 4A. Data for 24-h and 72-h groups in Fig. 3 are plotted in Fig. 4B. Slopes were 1.024 (r^2^ = 0.992) for A and 0.819 (r^2^ = 0.997) for B. Data are derived from 12 independent experiments.

Fig. 4 shows the results of correlation analysis between the PCR-Invader-derived total counts and viable counts. Both counts on the non-structured surface (control) exhibited a high correlation, suggesting that the Invader PLUS method correlated with the viable cell count (Fig. 4A). Both counts in the experimental group, having a residual structure, also exhibited a high correlation (Fig. 4B). This suggests that the amount of secondarily adhered *S. mutans* increased according to the volume of residual biofilm structure. Slopes were 1.024 (r^2^ = 0.992) for the control group and 0.819 (r^2^ = 0.997) for the experimental group.

## Discussion

Mechanical removal of dental biofilm with adequate oral hygiene procedures is regarded as an essential approach for the control of mature oral biofilms [41]. Antimicrobial measures such as topical antiseptics, local drug delivery and systemic antibiotics, have proven effective as complements to mechanical control [6, 38]. However, recent investigations have reported that no or little biofilm structure was removed when model oral biofilms were treated with ethanol [14, 16], chlorhexidine [14, 16, 17, 28], nisin [16, 27], glutaraldehyde [27], a quaternary ammonium compound [27], sodium lauryl sulfate [16], triclosan [16], cetylpyridinium chloride [16, 17] or essential oil [17]. Residual structures may negatively affect the oral environment by serving as a scaffold for the secondary adhesion of microorganisms, allowing re-development of the biofilm.

Based on the present results, our hypothesis that the residual biofilm structure after complete disinfection does not affect biofouling by planktonic cells is rejected. Three-dimensional reconstructed and cryosectioned images demonstrated stratified patterns of viable cells on the disinfected biofilm structure (Figs. 1 and 2). Moreover, secondary bacterial adhesion was significantly higher in the presence of residual structure (Fig. 3). Consistent with our findings is the observation that, after injection of *Porphyromonas gingivalis* in CHX-gluconate-disinfected *in vitro P. gingivalis* biofilms, the biovolume of the bacteria adherent to the residual biofilm structure was significantly higher when compared with saliva-coated wells [28]. Thus, the promotion of secondary adhesion by residual structure is not *S. mutans*-specific, but is a phenomenon that affects a variety of planktonic microorganisms.

In this study, a resin composite material was used to create the cryosectioned images in addition to the three-dimensional reconstructed images. Hydroxyapatite disks or enamel slices are generally used in order to reproduce the oral condition [34, 42, 43]. However, *S. mutans* biofilms were resistant to be removed from those specimens due to cohesive adhesion.

We used 15 minutes as the shorter period of adherence, mimicking the period of initial bacterial adhesion following professional mechanical tooth cleaning. A longer period of 4 hours was set under the assumption that the interval of dietary intake (about 4 hours) would be sufficient for biofilm formation following secondary adhesion. The secondary adhered cells increased in the existence of the residual structure following complete disinfection (Fig. 3). On 72-h disinfected structures, viable counts after 4 hours of secondary adhesion (Fig. 3C) were approximately ten times higher than those after 15 min of secondary adhesion (Fig. 3A). This increase may be caused by the growth-facilitative activity of the residual structure, in addition to increased adhesion.

The secondary adhesion of *S. mutans* to the residual structure may be attributed to glucan-dependent aggregation and cell-cell aggregation. The binding of *S. mutans* to glucans on the biofilm surface is mediated by cell-associated glucosyltransferase enzymes and glucan-binding proteins [44]. In particular, glucan-binding protein C is cell wall-bound and serves as a cell-surface glucan receptor [45, 46]. PAc, which is a 190-kDa surface protein on *S. mutans*, participates in sucrose-independent adherence and self-aggregation between cells [47, 48]. PAc may also bind to the salivary agglutinin on the biofilm surface *in vivo*, enabling secondary adhesion.

In this study, there was a high correlation in the control group, with a slope of 1.024 (Fig. 4A). In the experimental group, if the volume of the residual structure did not affect the amount of the secondary adhered cells, least-squares curve-fitting analysis would not exhibit a noticeable correlation. However, this analysis exhibited a high correlation with a slope of 0.819 (Fig. 4B). These results indicate that the amount of adherent *S. mutans* increases with residual structure volume, thus supporting the notion that the residual structure promotes secondary adhesion.

The residual structure may not only cause adverse effects with regard to host response, but may also be pathogenic; even if the microorganisms in the biofilm are completely killed, various microbial components in the biofilm could play a role in disease pathogenesis [49]. It has been reported that the injection of dead components of *Enterococcus faecalis* into rats following mechanical aortic damage by a catheter produced endocarditic vegetation enriched with polymorphonuclear cells [30]. In addition, the remaining dental biofilm structure will absorb calcium and phosphate from saliva and/or crevicular fluid to form calculus [32]. Although the calculus surface may not in itself induce inflammation in the adjacent periodontal tissue, calculus is known to be a plaque retention factor.

In this study, we focused on the secondary adhesion to the residual structure following the complete disinfection. However, adverse reactions that may occur through the action of surviving microorganisms inside the biofilm should also be considered, as it is not always be possible to fully disinfect bacteria from dental biofilm using chemical approaches.

Induction of biofilm-mediated antimicrobial resistance may be another negative consequence of antimicrobial agents without biofilm detachment. The main factors that contribute to resistance include inefficient diffusion and degradation of antimicrobials within the biofilm matrix [18, 23, 50]. In such cases, the bacteria in deeper areas are exposed to antimicrobials at subminimal inhibitory concentrations (sub-MIC). This can induce biofilm formation *in vitro*, interfering with bacterial biofilm virulence expression [51–55] or through mechanisms involving the intercellular second messenger cyclic dimeric guanosine monophosphate [56]. It has also been reported that *S. mutans* exposed to one-half MIC of sodium fluoride and chlorhexidine gluconate form a dense biofilm with an extensive extracellular matrix, and show upregulated expression of genes related to biofilm formation [57].

Although chemical agents provide some benefits in terms of controlling oral biofilms, they have the limitation of leaving biofilm structures that may induce adverse reactions such as biofilm regrowth. Future strategies for the control of oral biofilms may therefore shift to the degradation and/or detachment of biofilm matrix.

## Supporting Information

S1 Fig RDR reactor. Biofilm reactor used in this study consists of a reactor vessel, a rotating wheel with a magnetic bar, a peristaltic pump and culture medium.
(TIF)

S2 Fig Correlation between PCR-Invader-derived total counts and viable counts after treatment with heat-inactive DNase I (A: control), DNase I (B) and sterilization (0CFU). These experiments were performed to validate Invader PLUS method. After preparing for 24-h-old biofilms, three types of treatments were applied. Each sample was divided into equal volumes for viable cell count before performing Invader PLUS assay.
(TIF)
