# Supplementary figures and images for: Residual Structure of Streptococcus mutans Biofilm following Complete Disinfection Favors Secondary Bacterial Adhesion and Biofilm Re-Development
Source: PLoS One. 2015 Jan 30;10(1):e0116647. doi: 10.1371/journal.pone.0116647 (PMC4312048; doi:10.1371/journal.pone.0116647)

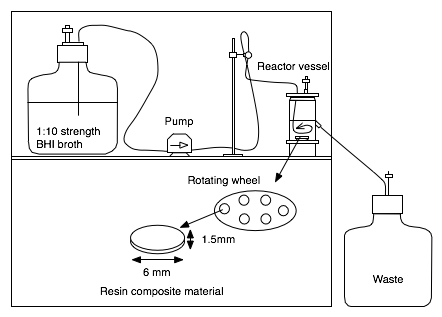

Supplement: S1 Fig — Biofilm reactor used in this study consists of a reactor vessel, a rotating wheel with a magnetic bar, a peristaltic pump and culture medium. (TIF) [file pone.0116647.s001.tif]

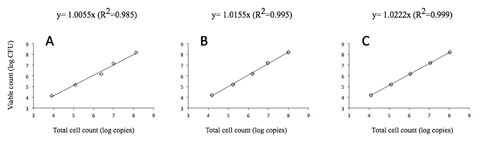

Supplement: S2 Fig — These experiments were performed to validate Invader PLUS method. After preparing for 24-h-old biofilms, three types of treatments were applied. Each sample was divided into equal volumes for viable cell count before performing Invader PLUS assay. (TIF) [file pone.0116647.s002.tif]
